# The induced and intrinsic resistance of *Escherichia coli* to sanguinarine is mediated by AcrB efflux pump

**DOI:** 10.1128/spectrum.03237-23

**Published:** 2023-12-01

**Authors:** Jian-Sheng Dai, Jian Xu, Hao-Jie Shen, Ni-Pi Chen, Bing-Qi Zhu, Zheng-Jie Xue, Hao-Han Chen, Zhi-Shan Ding, Rui Ding, Chao-Dong Qian

**Affiliations:** 1 College of Life Sciences, Zhejiang Chinese Medical University, Hangzhou, China; 2 College of Medical Technology, Zhejiang Chinese Medical University, Hangzhou, China; 3 School of Life Sciences, Anhui Medical University, Hefei, China; Brown University, Providence, Rhode Island, USA

**Keywords:** antimicrobial resistance, sanguinarine, antibiotic alternatives, *Escherichia coli*, efflux pump, AcrB

## Abstract

Sanguinarine-containing phytobiotic is becoming increasingly popularly utilized as antibiotic alternatives in animal production. However, their potential risks to the human health and environment remain poorly understood. The present study aims to evaluate the occurrence of drug resistance in *Escherichia coli* after treatment with sanguinarine and investigate the molecular basis associated with sanguinarine resistance. Repetitive exposure of *E. coli* to subinhibitory dose of this phytochemical selected for mutants within genes encoding AcrR or MarR, transcriptional regulators of the multidrug tripartite efflux pump AcrAB-TolC. Disruption of the gene *acrB* caused a 16-fold MIC decrease for sanguinarine against *E. coli* strains, while the susceptibility of *acrB*-deficient mutants to sanguinarine was restored in complemented strains with ectopically expressed *acrB*. Accumulation assays suggested that the high susceptibility of *acrB*-deficient mutants was a consequence of a decrease in drug efflux. Consistent with this, the antibacterial activity of sanguinarine against wild-type (WT) strains of *E. coli* was significantly strengthened by combining it with 1-(1-naphthylmethyl)-piperazine (NMP), a classical efflux pump inhibitor. In addition, the combination of *in silico* prediction, competitive inhibition assay, and functional analysis of AcrB variants led to the identification of sanguinarine as a CH3-preferring substrate of AcrB, which could partly explain the selectively enhanced antibacterial effect of NMP on sanguinarine.

**IMPORTANCE:**

The use of plant extracts is increasing as an alternative to synthetic compounds, especially antibiotics. However, there is no sufficient knowledge on the mechanisms and potential risks of antibiotic resistance induced by these phytochemicals. In the present study, we found that stable drug resistant mutants of *E. coli* emerged after repetitive exposure to sanguinarine and demonstrated that the AcrB efflux pump contributed to the emerging of induced and intrinsic resistance of *E. coli* to this phytochemical. Our results offered some insights into comprehending and preventing the onset of drug-resistant strains when utilizing products containing sanguinarine.

## INTRODUCTION

Antibiotic resistance has become a serious threat to human health. The extensive use of antibiotics in animal husbandry and veterinary medicine is a major driver of bacterial resistance (1). It has been estimated that approximately 73% of all antimicrobials sold worldwide are utilized in the production of food animals, particularly poultry and swine produced (2, 3). Antibiotics, as feed additives for disease prevention and growth promotion in food animals, specifically referred to as antibiotic growth promoters (AGPs), have made significant contributions to animal health, growth performance, and the overall productivity of the industry (4, 5). However, the heavy use of AGPs exacerbates the onset and spread of antibiotic resistance (2, 6). Concerns over the escalating prevalence of antibiotic-resistant bacteria have prompted many governments to prohibit the use of AGPs in animal production (7, 8). The prohibition has resulted in a significant reduction of resistance to various antibiotics in enterococci isolated from animal faeces, but it also exerts an adverse impact on animal production due to the increased prevalence of pathogenic infections (9, 10).

To mitigate the adverse impacts on animal production industries caused by the ban of in-feed antibiotics, a wide range of alternatives to AGPs have been proposed and developed. The classes of antibiotic alternatives used in animal production include probiotics, organic acids, prebiotics, antimicrobial peptides, enzymes, phytobiotics, and so on (8, 11). Among them, phytobiotics with antimicrobial properties exhibit the most similar performance to AGP and have demonstrated promising effects on animal production. Studies have shown that phytobiotics can effectively reduce pathogen proliferation, modulate intestinal microbiota, enhance feed intake, increase carcass quality and muscle yield, as well as stimulate the immune system (12 – 14). To date, there are various commercially available phytobiotics that are used to improve the health status and productivity parameters of animals, for example, Sangrovit, an herbal extract derived from the *Macleaya cordata* (15, 16). The phytobiotics have been widely utilized in animal feed for decades to promote livestock growth, which has been related mainly to their antimicrobial components.

Sanguinarine, one of the main active components of Sangrovit, is well known for its broad spectrum of pharmacological actions, especially its anti-infective effects (17, 18). Sanguinarine has demonstrated activity against *E. coli*, *Salmonella enterica*, *Listeria monocytogenes*, *Shigella flexneri*, *Vibrio parahaemolyticus,* and *Enterococcus faecalis* (19), which are common diarrhea-causing bacteria in animals. Sanguinarine also has inhibitory activity against parasites, including *Schistosoma mansoni*, *Dactylogyrus intermedius*, and *Toxocara canis* (20 – 22). Recently, Aljumaah et al. reported that dietary supplementation with sanguinarine-containing products could effectively maintain the growth performance and improve the meat characteristics of broilers challenged with *Salmonella typhimurium* (16). Due to their remarkable antimicrobial and growth-promoting properties, sanguinarine-containing products have gained increasing attentions as a potential alternative to antibiotics in livestock production (23).

Despite the antimicrobial activities of sanguinarine have been extensively studied, there is little information available regarding the development of drug resistance in microorganism after exposure to this phytochemical (24). This work therefore undertook to investigate the potential of *E. coli* for developing adaptive resistance to sanguinarine and elucidate the molecular basis underlying bacterial resistance.

## MATERIALS AND METHODS

### Bacterial strains and growth conditions

The strains and plasmids used are listed in Table 1. *E. coli* strains were grown aerobically at 37°C in Luria-Bertani (LB) broth or agar plates. Mueller-Hinton (MH) medium (Oxoid, UK) was used for drug susceptibility testing. The strains for pKD46 or pCP20 temperature-sensitive plasmid maintenance were incubated at 30°C. When needed, appropriate antibiotics for plasmid selection and maintenance were used at the following concentrations: ampicillin at 100 µg/mL and kanamycin at 50 µg/mL.

**TABLE 1:** Strains and plasmids used in this study

Strain or plasmid	Relevant characteristics	Source
*Escherichia coli s*trains		
35218	WT strain	ATCC
35218m	A spontaneous *acrB-*deficient mutant of *E. coli* ATCC 35218	This study
T1, T2, T3	Sanguinarine-induced *acrR-*deficient mutants of *E. coli* ATCC 35218	This study
T5, T6	Sanguinarine-induced *marR-*deficient mutants of *E. coli* ATCC 35218	This study
DH5α	WT strain; Host for cloning and plasmid isolation	Stratagene
Δ*acrB*	Derived from DH5α, in which the entire AcrB gene was deleted	This study
C35218m	35218m harboring pBBR-acrB to restore the ability of AcrB	This study
C∆*acrB*	Δ*acrB* carrying pBBR-acrB, which was used to complement the AcrB gene	This study
Triple mutant	35218m carrying pBBR-acrB-triple, which contains a mutant AcrB with A33W/T37W/N298W amino acids change	This study
Quadruple mutant	35218m with pBBR-acrB-quadruple, which contains a mutant AcrB with A33W/T37W/N298W amino acids change	This study
35218m-pBBR	35218m with an empty pBBR1MCS-2; used as a negative control strain	This study
Δ*acrB*-pBBR	Δ*acrB* with an empty pBBR1MCS-2; used as a negative control strain	This study
Plasmids		
pBBRMCS-2	Expression vector, Km^R^; oriRK2 mobRK2	(25)
pBBR-acrB	pBBR1MCS-2 derivative containing a wild type *acrB* gene cloned into HindIII and XbaI	This study
pBBR-acrB- triple	pBBR-acrB derivative carrying a mutant *acrB* with A33W/T37W/N298W amino acids change; Km^R^	This study
pBBR-acrB- quadruple	pBBR-acrB-triple derivative carrying a mutant *acrB* with A33W/T37W/ A100W/N298W amino acid change; Km^R^	This study
pKD46	Red recombinase expression plasmids, Amp^R^	(26)
pKD4	λRed template plasmid with a FRT-Km cassette; Amp^R^, Km^R^	(26)
pCP20	λRed helper plasmid; FLP^+^, Amp^R^,Cm^R^	(26)

### Chemicals

Sanguinarine (>98%) was purchased from Shanghai Macklin Biochemical Co., Ltd (China). Other chemicals and antibiotics were purchased from Aladdin Bio-Chem Technology Co., Ltd (Shanghai, China), unless otherwise stated. All agents were dissolved in Dimethyl sulfoxide (DMSO) and then diluted to ensure a final DMSO concentration of <2% (vol/vol).

### Drug susceptibility assay by MIC

The minimum inhibitory concentration (MIC) values were determined by using the broth microdilution method (27). The MIC was defined as the lowest concentration at which no visible bacterial growth was observed after incubation at 37°C for 20 h. To assess the combined effect, we utilized a checkerboard assay to determine the MIC of each agent alone and in combination, with subsequent analysis conducted via the fractional inhibitory concentration index (FICI) model (28).

### Mutant selection

Stable sanguinarine-resistant mutants of *E. coli* ATCC 35218 were isolated from LB cultures through repetitive exposure to a subinhibitory concentration of sanguinarine. The overnight culture of the WT *E. coli* ATCC 35218 was diluted to a ratio of 1:100 into 5 mL fresh LB broth and subsequently incubated in the presence of 1/2 MIC (8 µg/mL) of sanguinarine for 24 h. This bacterial suspension was then diluted 1:100 into 5 mL fresh LB broth containing the same concentration (8 µg/mL) of sanguinarine and incubated for 24 h. This procedure was repeated 10 times. After the 10th step, 100 µL aliquots were spread on plates containing 64, or 128 µg/mL of sanguinarine. Following the incubation period, single colonies were randomly selected, purified by streaking on drug-free LB plates, and their susceptibility to sanguinarine was measured by MIC testing.

A single-step selection procedure for isolating clones resistant to sanguinarine was also performed. Briefly, a 3 × 10^9^ CFU/mL inoculum of WT strain was spread onto LB plates containing different concentrations (1–8× MIC) of sanguinarine. All plates were incubated at 37°C for 48 h. Individual colonies were selected and streaked on LB agar without sanguinarine. The drug susceptibility assay was then performed.

### Whole genome sequencing and analysis

Genomic DNA was extracted from *E. coli* strain using the ZR Fungal/Bacterial Genomic DNA ExtractionKit (Zymo Research Corp, Orange, CA, United States) in accordance with established protocols. The genome sequencing was performed by Sangon Biotech (Shanghai, China) using the Illumina HiSeq sequencing platform. The quality of the high-throughout sequence data was assessed by FastQC. The quality control-filtered paired-end reads were mapped onto the genome sequence of *E. coli* ATCC 35218 (https://genomes.atcc.org/genomes/92c302c2e2f34245) using the Burrows-Wheeler alignment tool. The genome coverages for all strains were more than 200×. The consensus sequences were then compared to detect single nucleotide polymorphism (SNP) and indel differences by SAMtools and Genome Analysis Toolkit. The draft genomes of WT 35218 and its derived strains have been deposited in the NCBI database under SRA number (SUB 13190539).

### RT-PCR detected gene expression

The levels of expression for *marA*, *acrA,* and *acrB* were analyzed by real-time quantitative PCR (qPCR) as previously described (29, 30). Bacterial cells were grown overnight, diluted 1:100 in fresh LB, and grown for 3 h to about 0.3 OD_600_. RNA was extracted from each culture by Bacteria Total RNA Isolation Kit (Sangon biotech, China) and then reverse transcribed to cDNA using oligo(dT) primer. The obtained cDNA was then quantified in a qTOWER 3.0 system (Analytik Jena, Jena, Germany) using Thermo SYBR Select Master Mix. The 16S rRNA gene was used as internal standard. The primes used were listed in Table S1.

### Construction of the AcrB gene deletion mutant

The isogenic *acrB*-deficient mutant of *E. coli* DH5α was constructed by using Red recombination system as previously described (26). The kanamycin-resistance cassette gene flanked by 50 base pairs homology arms located upstream and downstream of the *acrB* gene was PCR amplified from pKD4 using primers acrB-P1/acrB-P2 (Table S2). The PCR product was purified via gel electrophoresis and transformed into competent cells of *E. coli* DH5α containing plasmid pKD46. Recombinants were selected by plating on LB agar containing kanamycin and confirmed by PCR amplification and DNA sequencing using primers acrB-H1/acrB-H2 (Table S2). The pKD46 plasmid was cured from recombinants by nonselective growth at 42℃. The kanamycin-resistance cassette was eliminated by transforming plasmid pCP20 and selecting the ampicillin and kanamycin-susceptibility strain, which was named as Δ*acrB*.

### Complementation of the *acrB* mutant

For functional complementation of the *acrB*-deficient strains, the *acrB* gene was amplified from chromosomal DNA of DH5α using primers pBBR-acrB-F/pBBR-acrB-R (Table S3) and inserted it into the HindIII and SacI sites of the low copy number plasmid pBBR1MCS-2 (25) using One Step Seamless Cloning kit (Genesand, China). The recombinant plasmid was transformed into clone strain *E. coli* DH5α. The transformants were spread on LB plates containing kanamycin at 37℃, and the resulting strains were then selected and confirmed by PCR using two pairs of primers: pBBR-acrB-1/pBBR-acrB-2 (Table S3) and pBBR-acrB- YZ-F/pBBR-acrB-YZ-R (Table S3). After further confirmed by DNA sequencing, the recombinant plasmid pBBR-acrB and empty plasmid pBBR1MCS-2 were transformed into 35218m and Δ*acrB* to generate strains C35218m, CΔ*acrB*, 35218m-pBBR, and Δ*acrB-*pBBR respectively.

### Site-directed mutagenesis

The AcrB triple (A33W/T37W/N298W) and quadruple (A33W/T37W/A100W/N298W) mutants were constructed by PCR-based site-directed mutagenesis as previously described (31). Briefly, the plasmid pBBR-acrB, carrying the WT sequence of *acrB* was used for the site-directed mutagenesis. The entire plasmid was amplified with primers listed in Table S4. The resultant products were transformed into DH5α after treatment with DnpI to eliminate interference from parent template. The mutants were validated (the validation primes were listed in Table S5) by sequencing and transformed into 35218m.

### Sanguinarine accumulation assay

The intracellular accumulation of sanguinarine was assessed by using the autofluorescence characteristics of sanguinarine measured with a spectrofluorometer (32). Briefly, an overnight culture of *E. coli* was inoculated in LB broth and incubated to mid-log phase. The cultures were then diluted to OD_600_ = 0.5 with fresh LB broth and treated with different concentration of sanguinarine or 1.6% DMSO for 1 h. The bacterial cells were harvested by centrifugation (8000 g, 5 min), washed twice with 1× potassium phosphate buffer (PBS) (pH 7.4), and resuspended with the same buffer. The fluorescence of samples was measured with a FLUOstar Omega Microplate Reader (BMG Labtech, Oenburg, Germany) at excitation and emission wavelengths of 485 and 590 nm, respectively.

### Competitive efflux inhibition assay

The efflux pump competitive inhibition test was assessed using ethidium bromide (EB) as described earlier with slight modifications (31). In brief, overnight cultures of *E. coli* were diluted 1:100 and then incubated at 37°C until logarithmic growth phase. Bacterial cells were harvested by centrifugation, washed twice with Efflux Buffer (100 mM potassium phosphate, 5 mM MgSO4, pH 7.5), and resuspended to final OD_600_ of 5. The cells were then exposed to 10 µM EB in presence of different concentration of sanguinarine (0, 1/4 × MIC, 1/2 × MIC, 1 × MIC). A control group was established by treating bacterial cells with the same concentration of sanguinarine to help eliminate the fluorescence interference of sanguinarine. The fluorescence intensity was monitored with excitation wavelength at 530 nm and emission wavelength at 600 nm.

### Molecular docking

Molecular docking calculations were performed for sanguinarine on *E. coli* AcrB (PDB ID: 4D × 5) using AutoDock Vina (33). Docking was operated within a rectangular search space of size 126 Å by 126 Å by 126 Å enclosing the protein AcrB. The exhaustiveness parameter was set to 104 (13 times the default 8). Protein and ligand input files in PDBQT format were prepared with AutoDock Tools.

## RESULTS AND DISCUSSION

### Repetitive exposure to sanguinarine selected for mutations exhibiting cross-resistance to sanguinarine and other antimicrobial agents

Emergence of strains exhibiting increased resistance to plant-derived antimicrobials has been observed following exposure to several phytochemicals. It remains unclear whether sanguinarine treatment may also induce heightened bacterial resistance. To gain information about sanguinarine resistance in *E. coli*, we induced bacterial resistance to this compound by using two selection procedures outlined in our methods section. We failed to obtain stable sanguinarine-resistant mutants by single-step selection. However, sanguinarine-resistant mutants of *E. coli* ATCC 35218 were obtained by repetitive exposure to subinhibitory doses of this agent for 10 days. Five colonies, designated as T1, T2, T3, T5, and T6, were randomly isolated. The MICs of these mutants were determined after three rounds of subculturing on drug-free medium. As shown in Table 2, the MIC of WT 35218 was 16 µg/mL of sanguinarine, whereas the derivative strains showed an increase in resistance, with MICs of 32–64 μg/mL of this phytochemical. The MICs of these resistant mutants were also found to be higher than those of WT 35218 against thymol, carvacrol, and chelerythrine, the latter of which is a structural analog of sanguinarine. In addition, an increase in the resistance of derivative strains to chloramphenicol, tetracycline, erythromycin, ciprofloxacin, but not kanamycin and polymyxin B was detected with regard to the WT strains. It should be noted that a 2-fold difference in a MIC value is usually not considered significant due to methodological limitations. However, given that this change in MIC was repeatedly observed, the reduced sensitivity of these mutants to sanguinarine and other compounds is plausible.

**TABLE 2:** MICs of sanguinarine and other compounds against *E. coli* ATCC 35218 and its derivative strains

Compound	MIC (μg/mL)					
	WT 35218	T1	T2	T3	T5	T6
Sanguinarine	16	32	32	32	64	64
Chelerythrine	8	16	16	16	64	64
Carvacrol	256	512	512	512	1,024	1,024
Thymol	512	1,024	>1,024	1,024	>1,024	>1,024
Erythromycin	64	128	128	128	128	128
Chloramphenicol	256	512	512	512	1,024	512
Tetracycline	4	8	8	8	32	16
Ciprofloxacin	<0.0156	0.0156	0.0156	0.0156	0.0156	0.0156
Kanamycin	2	2	2	2	2	2
Polymyxin B	2	2	2	2	2	2

### Mutations in *acrR* and *marR* were found in sanguinarine-resistant strains

The genomes of sanguinarine-resistant mutants were compared to that of the parental strain in order to identify genetic events associated with the resistant phenotype. Computer analysis of interstrain differences between the assembled genomes of WT and mutant strains revealed four potential single SNPs and 2 indels in six coding regions, which were annotated as *lon*, *acrR*, *marR*, *sfsA*, *hyaB*, and BGPOLHDB_00043 (Table 3), respectively. Subsequent confirmation of each variation was achieved through PCR amplification and sequencing. The mutation of gene BGPOLHDB_ 00043 was only found in mutant strain T2.

**TABLE 3:** Single nucleotide polymorphisms (SNP) and insertions (Ins) in mutant stains of *E. coli* ATCC 35218 compared to the WT strain

Genome position	Mutant strains	Gene	Mutation	Change	Information
46850	T2	BGPOLHDB_00043	Ins: +TGAACT	Ala246_Arg247 ins Val His	Uncharacterized protein conserved in bacteria
3625504	T1, T2, T3, T5, T6	*lon*	SNP: C2042T	Gly 681 Asp	ATP-dependent Lon protease, bacterial type
3995089	T1, T2,T3	*sfsA*	Ins: +G	Frame shift	DNA-binding protein, stimulates sugar fermentation
3600571	T1, T2, T3	*acrR*	SNP: G88C	Stop gain	Transcriptional regulator
3104674	T5, T6	*hyaB*	SNP: G923A	Pro 308 Leu	Ni, Fe-hydrogenase I large subunit
2556710	T5, T6	*marR*	SNP: G363A	Stop gain	Transcriptional regulators

All derivative strains harbored a C to T transition at genome position 3625504 within *lon*, causing an amino acid change (Gly681Asp). The *lon* gene encoded an ATP-dependent protease that played an important role in maintaining protein homeostasis in prokaryotes. The Lon protease was responsible for the degradation of abnormal and misfolded proteins as well as regulatory proteins such as MarA and SoxS (34, 35). Earlier works found that antibiotic-resistant variants of bacteria often carried *lon* mutations (36), which did not directly provide intrinsic resistance, but promoted the evolution of antibiotic resistance (37).

Three derivative strains (T1, T2, and T3) also harbored a frameshift mutation in *sfsA* and a transversion (C to G) at genome position 89 within *acrR* resulting in very early termination of translation within the coding region. The SfsA has been identified as a DNA-binding protein with the ability to stimulate sugar fermentation (38), while *acrR* was reported to encode the local *acrAB* repressor (39). Mutations within the *acrR* have been shown to lead to *acrA* and *acrB* overexpression and are often responsible for low-level drug resistance of clinical isolates or laboratory-derived antibiotic-resistant mutants of Gram-negative bacteria (40).

Differently, a missense variant in *hyaB* and a C to T transition at genome position 361 within *marR* resulting in early termination of translation in the coding region were also presented in mutant strains T5 and T6. The gene *hyaB* was predicted to encode the large subunits of [NiFe]-hydrogenases (41), while *marR* has been identified as a regulatory gene involved in nonspecific resistance systems that conferring resistance to multiple antibiotics (42). MarR was a repressor protein that regulates expression of the Mar regulon via MarA. Multidrug resistance in clinical and veterinary isolates of Enterobacteriaceae bacteria was frequently associated with overexpression of MarA (43, 44), a transcription factor that upregulates the efflux pump AcrAB-TolC.

**Fig 1:**
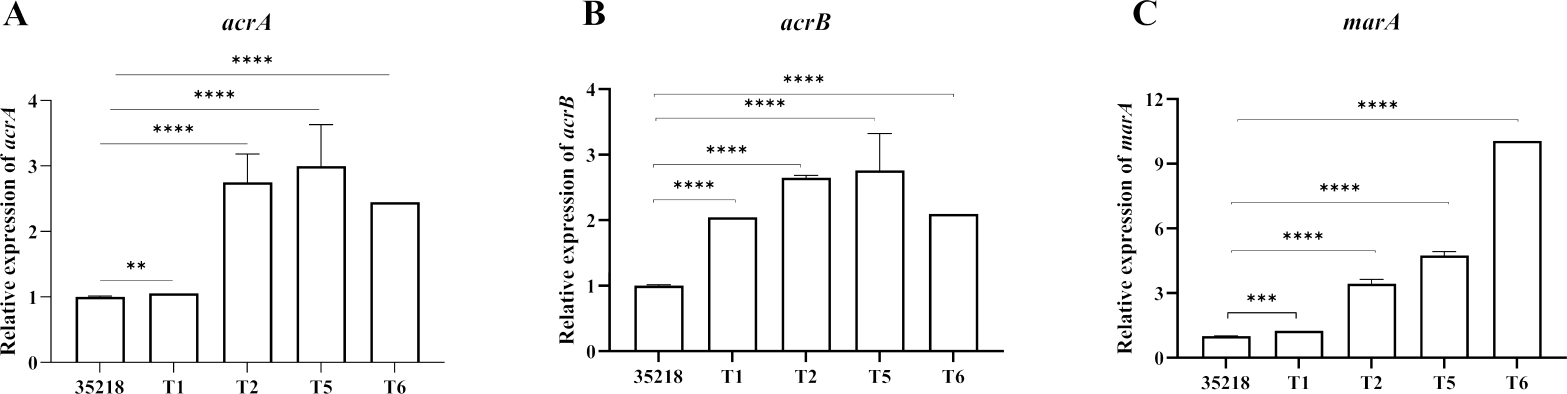
Effect of mutation in *acrR* or *marR* on the expression of the *acrA,* and *acrB,* and *marA*. The expression of the *acrA*, *acrB*, regulatory gene *marA*, as well as the control gene of 16 s rRNA were measured by RT–qPCR in the WT *E. coli* ATCC 35218 (35218) and its derived strains (**T1, T2, T5, and T6**). The results were presented as average   ± SEM (*n*  =  3) and shown normalized to the expression of each gene in the WT strain. Statistically significant differences between the WT and mutant strains are shown as **P  <  0.01, ***P  <  0.001, and ****P  <  0.0001.

Taken together, AcrR and MarR have been demonstrated to function as regulators that inhibit expression of the multidrug efflux pump AcrAB-TolC. The tripartite efflux pump is comprised of an inner membrane transport protein AcrB, a periplasmic fusion protein AcrA, and an outer membrane channel protein TolC. Among these components, the AcrB protein plays a pivotal role in the activity of the whole efflux system by identifying and capturing compounds within the bacteria (45, 46). It is widely acknowledged that three-component pump AcrAB-TolC is constitutively expressed and mainly responsible for the induced multidrug resistance in *E. coli* (47). Thus, the overexpression of AcrAB-TolC pump resulting from mutations in *acrR* and *marR* might mainly account for the increased resistance observed in the phenotypic characterization of mutants T1-3 and T5-6, respectively. To corroborate this, we found that the mRNA level of *acrA*, *acrB*, and *marA* was significantly higher in the mutants than that in their parent strain of *E. coli* ATCC 35218 (Fig. 1). While there are currently no published studies exploring the correlation between mutations in the genes *sfsA*, *hyaB*, or BGPOLHDB_00043 and bacterial resistance, the physiological significance of these genetic alterations is worthy of further investigation.

### Sanguinarine acted as a good substrate of the AcrB efflux pump in *E. coli*

Genetic variants of *E. coli*-resistant mutants after exposure to sanguinarine suggested that this compound may be a potential substrate of AcrAB-TolC. To verify this suggestion, antibacterial assays using pump-defective mutants were necessary to determine the impact of sanguinarine on strains lacking functional AcrAB-TolC efflux pump. Coincidentally, a spontaneous mutation arose in cultures of the WT 35218 strain during serial passage at 37°C. The mutant strain, designated as 35218m, exhibited heightened susceptibility to sanguinarine with a MIC of 1 µg/mL. Whole-genome sequence analysis revealed 11 potential mutations in 35218m, including nine single SNPs, two insertions in intergenic regions, and one large deletion (Table 4). Among them, the deletion mutation was predicted to result in very early termination of translation in a coding region that was annotated as multidrug efflux pump subunit AcrB. Therefore, we speculated that the loss of AcrB function in 35218m was the main factor contributing to increased sensitivity of this strain to sanguinarine compared to WT 35218. Consistent with this supposition, the susceptibility of 35218m to sanguinarine was fully restored by genetic complementation with the WT *acrB* gene (Table 5). Similar to the previous report, the MICs of 35218m to AcrB’s substrates erythromycin, chloramphenicol, tetracycline, and EB were significantly decreased (MIC changes of ≥4 fold) with regard to the WT 35218.

**TABLE 4:** Single nucleotide polymorphisms (SNP), insertions (Ins), and deletion (Del) in 35218m compared to the WT strain of *E. coli* ATCC 35218

Genome position	Gene	Mutation	Change	Information
386531	*hslO*	SNP: P245T	Pro24 Thr	33 kDa chaperonin
1509988	BGPOLHDB_01435	SNP: A363D	Ala133Asp	Hypothetical protein
1627044	*sixA-yfcV*	Ins: +T	Intergenic region	No
2017321	BGPOLHDB_01887	SNP: I120T	Ile120Thr	Hypothetical protein
2017342	BGPOLHDB_01887	SNP: V2042A	Val127Ala	Hypothetical protein
2282536	*ptrB*	SNP: P207L	Pro207Leu	Protease 2
2700395	BGPOLHDB_02513-BGPOLHDB_02514	Ins: +A	Intergenic region	No
3142114	*pncB*	SNP: L212I	Leu212Ile	Nicotinate phosphoribosyltransferase
3602758- 3602769	*acrB*_2	Del: AAATCCTGCTG	Stop gain,	Multidrug efflux pump subunit AcrB
4007838	*panC*	SNP: D220W	Arg220Trp	Pantothenate synthetase
4821698	*argC-argE*_2	SNP: A to G	Intergenic region	No
5105626	*yidZ*	SNP: A38T	Ala38Thr	HTH-type transcriptional regulator YidZ

**TABLE 5:** MICs of sanguinarine and other antimicrobial agents against *E. coli* strain with or without wild type *acrB* gene

Compound	MIC (μg/mL)					
	WT 35218	35218m	C35218m	DH5α	∆*acrB*	C∆*acrB*
Sanguinarine	16	1	16	16	1	16
Tetracycline	2	0.5	2	2	0.5	2
Erythromycin	64	4	32	128	8	64
Chloramphenicol	256	32	128	8	1	4
Ethidium bromide	128	16	128	128	1	64
Polymyxin B	2	2	2	1	1	1
Carvacrol	256	256	128	256	128	256
Thymol	512	512	512	512	256	256

To further clarify the relationship between the role of AcrB pump in antibacterial activity of sanguinarine against *E. coli*, we constructed an *acrB*-deleted mutant of *E. coli* DH5α (Δ*acrB*) together with its complemented strain (CΔ*acrB*), and determined the MICs of them to sanguinarine and other antimicrobial agents (Table 5). Compared to the parental strain, the *acrB*-defective mutant strain exhibited a significant increase in susceptibility (≥ 4 fold) to erythromycin, chloramphenicol, tetracycline, and EB. In contrast, the deletion of *acrB* gene did not affect the susceptibility of *E. coli* to polymyxin B, which is a poor substrate of AcrB (48). These results were consistent with the previous report (48, 49) and suggested that the function of AcrB pump has been disrupted successfully in the mutant strain of *E. coli* DH5α. As expected, the deletion of *acrB* resulted in a 16-fold reduction in MIC for sanguinarine. The MIC level of the mutant strain to sanguinarine was restored in the complemented strain (C∆*acrB*), indicating sanguinarine was a good substrate of the AcrAB-TolC efflux pump. It was interesting to note that the disruption of *acrB* had no or a slight impact on the MIC of *E. coli* to carvacrol or thymol. This might be due to that the drugs with smaller molecular weight could penetrate the outer membrane barrier rapidly through porin channels (45).

### AcrB inactivated increased the accumulation of sanguinarine in *E. coli*

**Fig 2:**
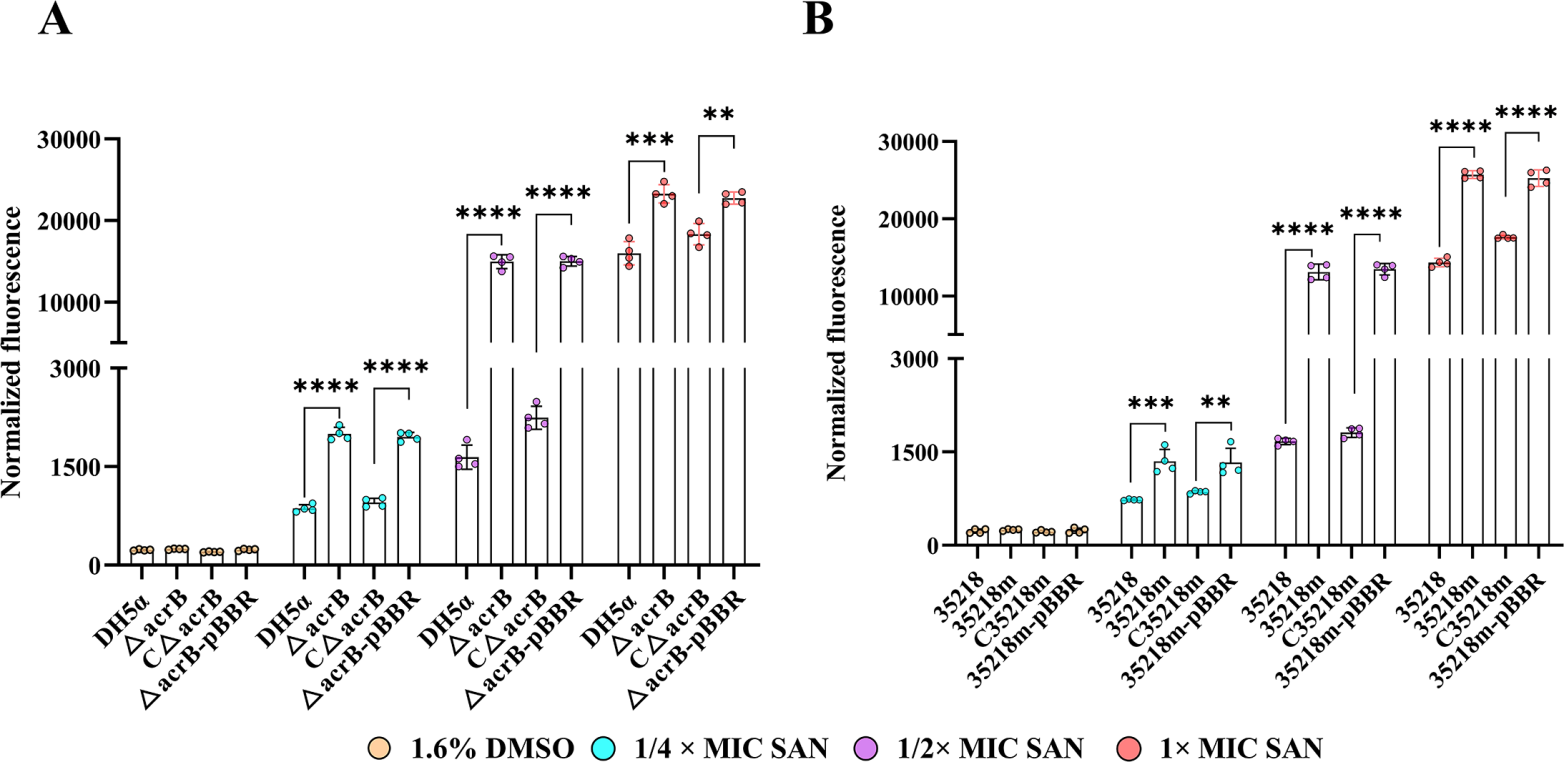
Intracellular accumulation of sanguinarine in *E. coli* with or without WT acrB *gene*. A, normalized fluorescence of intracellular sanguinarine in *E. coli* DH5α, Δ*acrB*, CΔ*acrB,* and Δ*acrB*–pBBR. B, normalized fluorescence of intracellular sanguinarine in *E. coli* ATCC 35218, 35218m, C35218m, 35218m-pBBR. The fluorescence intensity was measured at 1 h after sanguinarine treatment and normalized with OD_600_ values in order to eliminate the effect of cell growth. Data are presented as mean ± SD of four independent experiments. Amber color cycle, 1.6%(v/v) DMSO; Cyan cycle, 4 µg/mL SAN; Magenta color cycle, 8 µg/mL SAN; Orange red color cycle, 16 µg/mL SAN. Statistical significance was determined by a two-way ANOVA (**P < 0.01, ***P < 0.001, ****P < 0.0001). SAN, sanguinarine.

To understand whether increased susceptibility is a consequence of a decrease in drug efflux in *acrB*-deficient *E. coli* strain, we carried out accumulation assays with sanguinarine by using its autofluorescence characteristics (32). As shown in Fig. 2A, Δ*acrB* mutant exhibited significantly higher fluorescent intensity than the parental DH5α strain and CΔ*acrB* mutant after treatment with different concentrations of sanguinarine, suggesting the involvement of the AcrB pump in the efflux of this phytochemical. Similarly, the accumulation level of sanguinarine in the 35218m was markedly higher than that in the WT 35218 strain and complemented 35218m mutant (Fig. 2B). The ability to restore intracellular levels of sanguinarine to the WT levels through trans-complementation of Δ*acrB*/35218m with a full-length AcrB gene supported the role of AcrB pump in the efflux of sanguinarine.

### The antibacterial activity of sanguinarine against WT strain of *E. coli* was enhanced by NMP

Considering the critical role of AcrB pump in innate resistance to sanguinarine in *E. coli*, the effect of the classical efflux pump inhibitors Phe-Arg β-naphthylamide (PAβN) and 1-(1-naphthylmethyl)-piperazine (NMP) on the antimicrobial activity of this phytochemical was assessed by checkerboard assays (Table 6). In both WT strains (35218 and DH5α), PAβN at 20 µg/mL significantly reduced the MIC of erythromycin 16-fold, but it had no effect on the MICs of sanguinarine and EB, even when the concentration of PAβN was raised to 50 µg/mL (data not shown). In contrast, NMP at a concentration of 50 µg/mL had limited effects on the MICs of erythromycin in WT strains of *E. coli*, but it significantly reduced the MICs of sanguinarine and EB against them by 4-fold. In parallel assays, NMP marginally decreased the MICs of sanguinarine in AcrB mutants by a factor of two, suggesting that NMP increased sanguinarine’s antibacterial action against WT *E. coli* at least in part through the inhibition of AcrB function.

**TABLE 6:** The MICs of sanguinarine in combination with pump inhibitor

Compound	PAβN^*a*^	MIC (μg/mL)					MIC (μg/mL)				
		35218	35218m	DH5α	∆*acrB*	NMP^*b*^	35218	35218m	DH5α	∆*acrB*	
Sanguinarine	−	16	1	16	1	−	16	1	16	1	
	+	16	2	16	4	+	4	0.5	4	0.5	
Ethidium bromide	−	128	16	128	1	−	128	16	128	1	
	+	128	16	128	4	+	32	8	32	1	
Erythromycin	−	64	4	128	8	−	64	4	128	8	
	+	4	0.25	8	0.5	+	32	0.5	128	4	

^*a*^ 20 μg/mL.

^*b*^ 50 μg/mL.

### Sanguinarine seemed to be a channel 3 preferring substrate of AcrB

The crystallographic structure of AcrB from *E. coli* was solved as a symmetrical trimer (50) and subsequently as an asymmetric trimer (51, 52), in which each protomer adopted a distinct conformation: loose, tight, and open (or: access, binding, and extrusion). The protomers underwent a series of structural changes from access to binding, then extrusion states during substrate transport. Two primary substrate binding regions, the access pocket (AP) and the deep binding pocket (DBP), were identified in the periplasmic porter domain of AcrB. In addition, multiple substrate channels (CH1- 4) have been described, with putative entrances from the outer leaflet of the inner membrane (CH1 and 4) or the periplasm space (CH2 and 3) toward the two drug-binding pockets (53).

**Fig 3:**
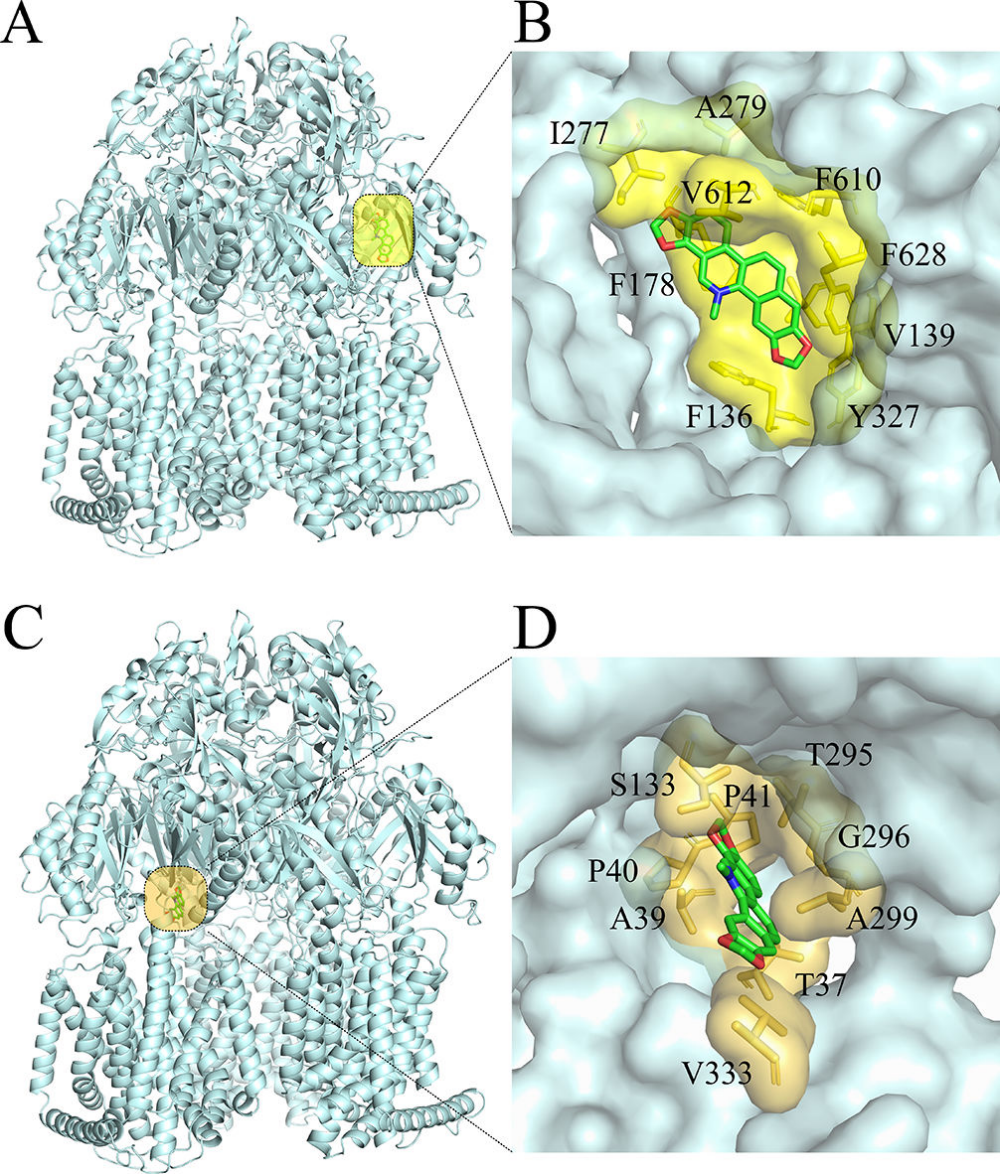
Conformations of the most stable binding modes of sanguinarine (shown as sticks: carbon, green; oxygen, red; nitrogen, blue) within the distal binding pocket (DBP) and channel 3 (CH3) of AcrB (pale cyan, shown as cartoon or surface). The images were created by PyMol. The yellow (**A**) and yellow orange (**C**) box indicated the binding site in DBP and CH3, respectively. B and D were zoomed images to show the residues (shown as surface and sticks) of AcrB have hydrophobic contacts with sanguinarine.

To understand the molecular basis of sanguinarine interaction with the AcrB pump of *E. coli*, the molecular docking was performed. The binding poses were evaluated and ranked using a scoring function. A large number of high-affinity poses were found within the distal binding pocket of the tight protomer of AcrB. The top ranked binding pose of sanguinarine was displayed in Fig. 3. According to the docking simulation, sanguinarine formed the hydrophobic interaction with the amino acid residues F136, V139, F178, I277, A279, Y327, F610, and V612 in the tight protomer DBP. Further inspection of the blind docking results revealed that sanguinarine is also bound to the channel 3 (CH3) of the loose protomer. The benzophenanthridine skeleton of sanguinarine penetrated deepest into CH3 entry gates of loose protomer in AcrB, making contact with the residues T37, A39, P40, P41, S133, T295, G296, A299, and V333 through hydrophobic interaction, suggesting sanguinarine might be a CH3-preferring substrate of AcrB.

**Fig 4:**
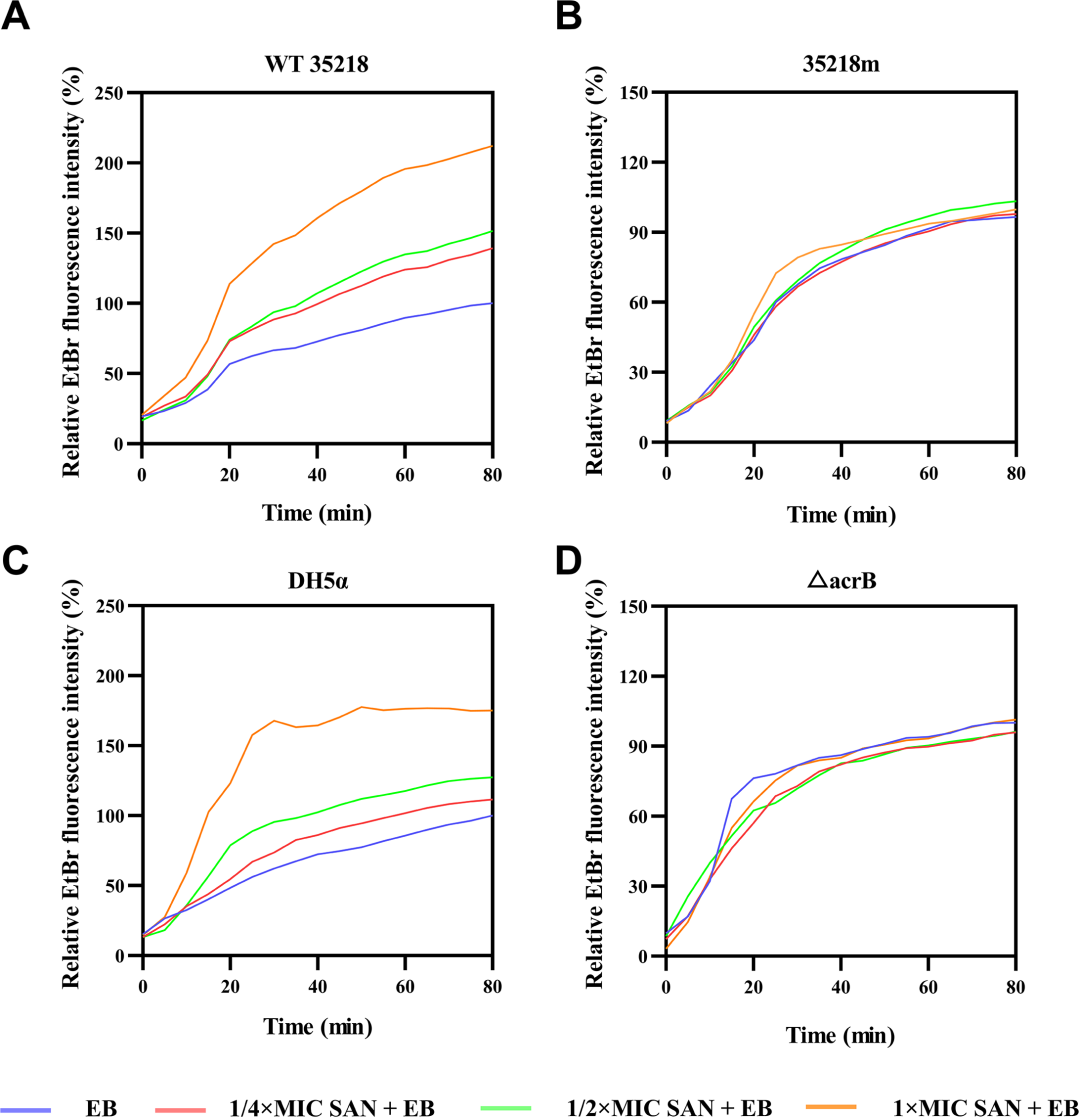
The competitive inhibition of sanguinarine on ethidium bromide efflux. Fluorescence intensity of ethidium bromide in *E. coli* ATCC 35218 (**A**) 35218m (**B**) *E. coli* DH5α (**C**) and Δ*acrB* (**D**) treated with different concentration sanguinarine (1/4 × MIC,1/2 × MIC, 1 × MIC). The vertical axis displays the percentage of fluorescence relative to the accumulation of EB treated alone in *E. coli* cells, indicating the degree of cellular uptake. The experiment was repeated three times with similar results, and one representative result is shown. SAN, sanguinarine; EB, ethidium bromide.

It has been reported that two CH3-preferring drugs, such as EB and benzalkonium chloride, were competing in the efflux cycle (31). To determine whether sanguinarine acted as a CH3-preferring compound, we measured the competitive inhibition effect of sanguinarine on the EB efflux in *E. coli* strains. As shown in Fig. 4, sanguinarine inhibited the EB efflux from WT 35218 and DH5α but not *acrB*-defective mutants in a concentration-dependent manner. In line with this, the combinations of sanguinarine with EB showed synergistic antibacterial activity against WT strains but not AcrB mutants of *E. coli* (Table 7). As a negative control, erythromycin, a CH2 preferring drug, did not enhance the antibacterial activity of sanguinarine against the tested WT strains.

**TABLE 7:** The MICs of sanguinarine in combination with other antimicrobial agents

Strains	Compound		MIC (μg/mL)		FIC^*a*^		FICI	Category^*b*^
	A	B	A	B	FIC A	FIC B		
35218	Sanguinarine	Ethidium bromide	16	128	4/16	32/128	0.5	Synergy
	Sanguinarine	Erythromycin	16	64	8/16	32/64	1	Additive
DH5α	Sanguinarine	Ethidium bromide	16	128	4/16	4/128	0.28	Synergy
	Sanguinarine	Erythromycin	16	128	8/16	128/128	1.5	No interaction
35218m	Sanguinarine	Ethidium bromide	1	16	0.5/0.5	8/16	1.5	No interaction
∆*acrB*	Sanguinarine	Ethidium bromide	1	1	0.5/1	0.5/1	1	Additive

^*a*^ FIC A = MIC of drug A in combination/MIC of drug A alone; FIC B = MIC of drug B in combination/MIC of drug B alone.

^*b*^ FICI categories: ≤ 0.5, synergistic; > 0.5 to ≤ 1, additive; > 1 to < 4, no interaction; ≥ 4, antagonism.

The amino acid residues (Ala33, Thr37, Ala100, and Asn298) situated in the vicinity of the entrance of CH3 have been reported to have a unique influence on the efflux of CH3 preferring compounds (31). When the A100W mutation was introduced to the triple mutant (A33W/T37W/N298W), the CH3 preferring substrates were exported more efficiently by quadruple mutant (A33W/T37W/A100W/N298W) compared to the triple mutant, resulting a higher MIC of these compounds against quadruple mutant than triple mutant strain. As expected, the susceptibility of the quadruple mutant strain was decreased by 2-fold to sanguinarine compared to that of triple mutant strain (Table 8). A similar decrease in activity was observed for EB but not erythromycin. These results further suggested that sanguinarine was a CH3-preferring substrate of AcrB.

**TABLE 8:** The MICs of *acrB* triple mutant and quadruple mutant to sanguinarine, ethidium bromide, and erythromycin

Compound	MIC (μg/mL)			
	WT 35218	35218m	Triple	Quadruple
Sanguinarine	16	1	4	8
Ethidium bromide	128	16	32	64
Erythromycin	64	4	32	32

Recent studies indicated that AcrB substrates with varied physicochemical features utilized different channels, and that CH3-preferring drugs exhibited competitive transport with each other, but not with drugs that prefer the other channel (31, 54). Sanguinarine, similar to EB, is a planar aromatic cation with a relatively low molecular mass (<500  Da), which has been postulated to be characteristic of CH3 preferring substrates of AcrB (31). Although NMP and PAβN are both cationic, but not planar compounds, the naphthalene ring of NMP confers the molecular core of this molecule a flatter conformation than that presented in PAβN. The finding that sanguinarine prefers CH3 at least in part can explain the selective synergistic effect of NMP but not PAβN on the antibacterial activity of sanguinarine against *E. coli* containing a functional AcrB.

In conclusion, we isolated several stable mutant strains of *E. coli* displaying an increased MIC to sanguinarine after repetitive exposure to this phytochemical. Worryingly, these mutant strains also display enhanced resistance to antibiotics chloramphenicol, tetracycline, erythromycin, and phytochemicals thymol, and carvacrol. In agreement with these observations, we find that the AcrB efflux pump contributes to the induced and intrinsic resistance of *E. coli* to sanguinarine. Although further experiments are necessary to explore whether resistant mutants will be induced in human and animal by sanguinarine-containing products, our findings provide some insights into the understanding and preventing of the emergence of drug resistant strains when using the phytobiotics.

## Data Availability

The Illumina sequencing data for all mutants have been deposited to NCBI under SRA accession numbers SAMN34413090 to SAMN34413094.

## SUPPLEMENTAL MATERIAL

Tables S1 to S5 spectrum.03237-23-s0001.pdf All primers used in this research.

ASM does not own the copyrights to Supplemental Material that may be linked to, or accessed through, an article. The authors have granted ASM a non-exclusive, world-wide license to publish the Supplemental Material files. Please contact the corresponding author directly for reuse.

## References

[1] Van Boeckel TP , Glennon EE , Chen D , Gilbert M , Robinson TP , Grenfell BT , Levin SA , Bonhoeffer S , Laxminarayan R . 2017. Reducing antimicrobial use in food animals. Science 357:1350–1352. doi:10.1126/science.aao1495 28963240 PMC6510296

[2] Van Boeckel TP , Pires J , Silvester R , Zhao C , Song J , Criscuolo NG , Gilbert M , Bonhoeffer S , Laxminarayan R . 2019. Global trends in antimicrobial resistance in animals in low- and middle-income countries. Science 365:eaaw1944. doi:10.1126/science.aaw1944 31604207

[3] Peng Z , Hu Z , Li Z , Zhang X , Jia C , Li T , Dai M , Tan C , Xu Z , Wu B , Chen H , Wang X . 2022. Antimicrobial resistance and population genomics of multidrug-resistant Escherichia coli in pig farms in mainland China. Nat Commun 13:1116. doi:10.1038/s41467-022-28750-6 35236849 PMC8891348

[4] Dibner JJ , Richards JD . 2005. Antibiotic growth promoters in agriculture: history and mode of action. Poult Sci 84:634–643. doi:10.1093/ps/84.4.634 15844822

[5] Broom LJ . 2018. Gut barrier function: effects of (antibiotic) growth promoters on key barrier components and associations with growth performance. Poult Sci 97:1572–1578. doi:10.3382/ps/pey021 29462405

[6] Gonzalez Ronquillo M , Angeles Hernandez JC . 2017. Antibiotic and synthetic growth promoters in animal diets: review of impact and analytical methods. Food Control 72:255–267. doi:10.1016/j.foodcont.2016.03.001

[7] Castanon JIR . 2007. History of the use of antibiotic as growth promoters in European poultry feeds. Poult Sci 86:2466–2471. doi:10.3382/ps.2007-00249 17954599

[8] JianGuo Z . 2022. Application and development progress of alternatives to in-feed antibiotics. Feed industry magazine 43:1–6. doi:10.13302/j.cnki.fi.2022.09.001

[9] Wierup M . 2001. The Swedish experience of the 1986 year ban of antimicrobial growth promoters, with special reference to animal health, disease prevention, productivity, and usage of antimicrobials. Microb Drug Resist 7:183–190. doi:10.1089/10766290152045066 11442345

[10] Brown K , Uwiera RRE , Kalmokoff ML , Brooks SPJ , Inglis GD . 2017. Antimicrobial growth promoter use in livestock: a requirement to understand their modes of action to develop effective alternatives. Int J Antimicrob Agents 49:12–24. doi:10.1016/j.ijantimicag.2016.08.006 27717740

[11] Helmy YA , Taha-Abdelaziz K , Hawwas H-H , Ghosh S , AlKafaas SS , Moawad MMM , Saied EM , Kassem II , Mawad AMM . 2023. Antimicrobial resistance and recent alternatives to antibiotics for the control of bacterial pathogens with an emphasis on foodborne pathogens. Antibiotics (Basel) 12:274. doi:10.3390/antibiotics12020274 36830185 PMC9952301

[12] Lillehoj H , Liu Y , Calsamiglia S , Fernandez-Miyakawa ME , Chi F , Cravens RL , Oh S , Gay CG . 2018. Phytochemicals as antibiotic alternatives to promote growth and enhance host health. Vet Res 49:76. doi:10.1186/s13567-018-0562-6 30060764 PMC6066919

[13] Zeng Z , Zhang S , Wang H , Piao X . 2015. Essential oil and aromatic plants as feed additives in non-ruminant nutrition: a review. J Anim Sci Biotechnol 6:7. doi:10.1186/s40104-015-0004-5 25774291 PMC4359495

[14] Kuralkar P , Kuralkar SV . 2021. Role of herbal products in animal production - an updated review. J Ethnopharmacol 278:114246. doi:10.1016/j.jep.2021.114246 34052352

[15] Aljumaah MR , Alkhulaifi MM , Abudabos AM . 2020. In vitro antibacterial efficacy of non-antibiotic growth promoters in poultry industry. J Poult Sci 57:45–54. doi:10.2141/jpsa.0190042 32174764 PMC7063082

[16] Aljumaah MR , Suliman GM , Abdullatif AA , Abudabos AM . 2020. Effects of phytobiotic feed additives on growth traits, blood biochemistry, and meat characteristics of broiler chickens exposed to Salmonella typhimurium. Poult Sci 99:5744–5751. doi:10.1016/j.psj.2020.07.033 33142492 PMC7647753

[17] Croaker A , King GJ , Pyne JH , Anoopkumar-Dukie S , Liu L . 2016. Sanguinaria canadensis: traditional medicine, phytochemical composition, biological activities and current uses. Int J Mol Sci 17:1414. doi:10.3390/ijms17091414 27618894 PMC5037693

[18] Laines-Hidalgo JI , Muñoz-Sánchez JA , Loza-Müller L , Vázquez-Flota F . 2022. An update of the sanguinarine and benzophenanthridine alkaloids’ biosynthesis and their applications. Molecules 27:1378. doi:10.3390/molecules27041378 35209167 PMC8876366

[19] Miao F , Yang X-J , Zhou L , Hu H-J , Zheng F , Ding X-D , Sun D-M , Zhou C-D , Sun W . 2011. Structural modification of sanguinarine and chelerythrine and their antibacterial activity. Nat Prod Res 25:863–875. doi:10.1080/14786419.2010.482055 21491327

[20] Zhang S-M , Coultas KA . 2013. Identification of plumbagin and sanguinarine as effective chemotherapeutic agents for treatment of schistosomiasis. Int J Parasitol Drugs Drug Resist 3:28–34. doi:10.1016/j.ijpddr.2012.12.001 23641325 PMC3638872

[21] Wang G-X , Zhou Z , Jiang D-X , Han J , Wang J-F , Zhao L-W , Li J . 2010. In vivo anthelmintic activity of five alkaloids from Macleaya microcarpa (Maxim) Fedde against Dactylogyrus intermedius in Carassius auratus. Vet Parasitol 171:305–313. doi:10.1016/j.vetpar.2010.03.032 20413222

[22] Satou T , Akao N , Matsuhashi R , Koike K , Fujita K , Nikaido T . 2002. Inhibitory effect of isoquinoline alkaloids on movement of second-stage larvae of Toxocara canis. Biol Pharm Bull 25:1651–1654. doi:10.1248/bpb.25.1651 12499659

[23] Liu X , Liu Y , Huang P , Ma Y , Qing Z , Tang Q , Cao H , Cheng P , Zheng Y , Yuan Z , et al. . 2017. The genome of medicinal plant Macleaya cordata provides new insights into benzylisoquinoline alkaloids metabolism. Mol Plant 10:975–989. doi:10.1016/j.molp.2017.05.007 28552780

[24] Zhang Q , Zhang Z , Zhou S , Jin M , Lu T , Cui L , Qian H . 2021. Macleaya cordata extract, an antibiotic alternative, does not contribute to antibiotic resistance gene dissemination. J Hazard Mater 412:125272. doi:10.1016/j.jhazmat.2021.125272 33550129

[25] Kovach ME , Elzer PH , Hill DS , Robertson GT , Farris MA , Roop RM , Peterson KM . 1995. Four new derivatives of the broad-host-range cloning vector pBBR1MCS, carrying different antibiotic-resistance cassettes. Gene 166:175–176. doi:10.1016/0378-1119(95)00584-1 8529885

[26] Datsenko KA , Wanner BL . 2000. One-step inactivation of chromosomal genes in Escherichia coli K-12 using PCR products. Proc Natl Acad Sci U S A 97:6640–6645. doi:10.1073/pnas.120163297 10829079 PMC18686

[27] Clinical and laboratory standards Institute . 2018. M100 performance standards for antimicrobial susceptibility testing, 28th ed. CLSI, Wayne, PA.

[28] Sopirala MM , Mangino JE , Gebreyes WA , Biller B , Bannerman T , Balada-Llasat J-M , Pancholi P . 2010. Synergy testing by etest, microdilution checkerboard, and time-kill methods for pan-drug-resistant Acinetobacter baumannii. Antimicrob Agents Chemother 54:4678–4683. doi:10.1128/AAC.00497-10 20713678 PMC2976112

[29] Ruiz C , Levy SB . 2014. Regulation of acrAB expression by cellular metabolites in Escherichia coli. J Antimicrob Chemother 69:390–399. doi:10.1093/jac/dkt352 24043404 PMC3886929

[30] Linkevicius M , Anderssen JM , Sandegren L , Andersson DI . 2016. Fitness of Escherichia coli mutants with reduced susceptibility to tigecycline. J Antimicrob Chemother 71:1307–1313. doi:10.1093/jac/dkv486 26851608 PMC4830415

[31] Zwama M , Yamasaki S , Nakashima R , Sakurai K , Nishino K , Yamaguchi A . 2018. Multiple entry pathways within the efflux transporter AcrB contribute to multidrug recognition. Nat Commun 9:124. doi:10.1038/s41467-017-02493-1 29317622 PMC5760665

[32] Lu C , Zhang N , Kou S , Gao L , Peng B , Dai Y , Zheng J . 2022. Sanguinarine synergistically potentiates aminoglycoside‐mediated bacterial killing. Microb Biotechnol 15:2055–2070. doi:10.1111/1751-7915.14017 35318794 PMC9249330

[33] Trott O , Olson AJ . 2010. AutoDock Vina: improving the speed and accuracy of docking with a new scoring function, efficient optimization, and multithreading. J Comput Chem 31:455–461. doi:10.1002/jcc.21334 19499576 PMC3041641

[34] Tsilibaris V , Maenhaut-Michel G , Van Melderen L . 2006. Biological roles of the Lon ATP-dependent protease. Res Microbiol 157:701–713. doi:10.1016/j.resmic.2006.05.004 16854568

[35] Griffith KL , Shah IM , Wolf RE . 2004. Proteolytic degradation of Escherichia coli transcription activators SoxS and MarA as the mechanism for reversing the induction of the superoxide (SoxRS) and multiple antibiotic resistance (Mar) regulons. Mol Microbiol 51:1801–1816. doi:10.1046/j.1365-2958.2003.03952.x 15009903

[36] Nicoloff H , Perreten V , Levy SB . 2007. Increased genome instability in Escherichia coli lon mutants: relation to emergence of multiple-antibiotic-resistant (Mar) mutants caused by insertion sequence elements and large tandem genomic amplifications. Antimicrob Agents Chemother 51:1293–1303. doi:10.1128/AAC.01128-06 17220404 PMC1855481

[37] Nicoloff H , Andersson DI . 2013. Lon protease inactivation, or translocation of the lon gene, potentiate bacterial evolution to antibiotic resistance. Mol Microbiol 90:1233–1248. doi:10.1111/mmi.12429 24325250

[38] Takeda K , Akimoto C , Kawamukai M . 2001. Effects of the Escherichia coli sfsA gene on mal genes expression and a DNA binding activity of SfsA. Biosci Biotechnol Biochem 65:213–217. doi:10.1271/bbb.65.213 11272834

[39] Ma D , Alberti M , Lynch C , Nikaido H , Hearst JE . 1996. The local repressor AcrR plays a modulating role in the regulation of acrAB genes of Escherichia coli by global stress signals. Mol Microbiol 19:101–112. doi:10.1046/j.1365-2958.1996.357881.x 8821940

[40] Camp J , Schuster S , Vavra M , Schweigger T , Rossen JWA , Reuter S , Kern WV . 2021. Limited multidrug resistance efflux pump overexpression among multidrug-resistant Escherichia coli strains of ST131. Antimicrob Agents Chemother 65:e01735-20. doi:10.1128/AAC.01735-20 33468485 PMC8097430

[41] Forzi L , Sawers RG . 2007. Maturation of [NiFe]-hydrogenases in Escherichia coli. Biometals 20:565–578. doi:10.1007/s10534-006-9048-5 17216401

[42] Miller PF , Sulavik MC . 1996. Overlaps and parallels in the regulation of intrinsic multiple-antibiotic resistance in Escherichia coli. Mol Microbiol 21:441–448. doi:10.1111/j.1365-2958.1996.tb02553.x 8866468

[43] Praski Alzrigat L , Huseby DL , Brandis G , Hughes D . 2017. Fitness cost constrains the spectrum of marR mutations in ciprofloxacin-resistant Escherichia coli. J Antimicrob Chemother 72:3016–3024. doi:10.1093/jac/dkx270 28962020 PMC5890708

[44] Webber M , Buckley AM , Randall LP , Woodward MJ , Piddock LJV . 2006. Overexpression of marA, soxS and acrB in veterinary isolates of Salmonella enterica rarely correlates with cyclohexane tolerance. J Antimicrob Chemother 57:673–679. doi:10.1093/jac/dkl025 16492722

[45] Li X-Z , Plésiat P , Nikaido H . 2015. The challenge of efflux-mediated antibiotic resistance in Gram-negative bacteria. Clin Microbiol Rev 28:337–418. doi:10.1128/CMR.00117-14 25788514 PMC4402952

[46] Alenazy R . 2022. Drug efflux pump inhibitors: a promising approach to counter multidrug resistance in Gram-negative pathogens by targeting AcrB protein from AcrAB-TolC multidrug efflux pump from Escherichia coli. Biology (Basel) 11:1328. doi:10.3390/biology11091328 36138807 PMC9495857

[47] Okusu H , Ma D , Nikaido H . 1996. AcrAB efflux pump plays a major role in the antibiotic resistance phenotype of Escherichia coli multiple-antibiotic-resistance (Mar) mutants. J Bacteriol 178:306–308. doi:10.1128/jb.178.1.306-308.1996 8550435 PMC177656

[48] Rieg S , Huth A , Kalbacher H , Kern WV . 2009. Resistance against antimicrobial peptides is independent of Escherichia coli AcrAB, Pseudomonas aeruginosa MexAB and Staphylococcus aureus NorA efflux pumps. Int J Antimicrob Agents 33:174–176. doi:10.1016/j.ijantimicag.2008.07.032 18945595

[49] Ciusa ML , Marshall RL , Ricci V , Stone JW , Piddock LJV . 2022. Absence, loss-of-function, or inhibition of Escherichia coli AcrB does not increase expression of other efflux pump genes supporting the discovery of AcrB inhibitors as antibiotic adjuvants. J Antimicrob Chemother 77:633–640. doi:10.1093/jac/dkab452 34897478 PMC8865010

[50] Murakami S , Nakashima R , Yamashita E , Yamaguchi A . 2002. Crystal structure of bacterial multidrug efflux transporter AcrB. Nature 419:587–593. doi:10.1038/nature01050 12374972

[51] Murakami S , Nakashima R , Yamashita E , Matsumoto T , Yamaguchi A . 2006. Crystal structures of a multidrug transporter reveal a functionally rotating mechanism. Nature 443:173–179. doi:10.1038/nature05076 16915237

[52] Seeger MA , Schiefner A , Eicher T , Verrey F , Diederichs K , Pos KM . 2006. Structural asymmetry of AcrB trimer suggests a peristaltic pump mechanism. Science 313:1295–1298. doi:10.1126/science.1131542 16946072

[53] Kobylka J , Kuth MS , Müller RT , Geertsma ER , Pos KM . 2020. AcrB: a mean, keen, drug efflux machine. Ann N Y Acad Sci 1459:38–68. doi:10.1111/nyas.14239 31588569

[54] Tam H-K , Foong WE , Oswald C , Herrmann A , Zeng H , Pos KM . 2021. Allosteric drug transport mechanism of multidrug transporter AcrB. Nat Commun 12:3889. doi:10.1038/s41467-021-24151-3 34188038 PMC8242077

