# Supplementary material for: The induced and intrinsic resistance of Escherichia coli to sanguinarine is mediated by AcrB efflux pump
Source: Microbiol Spectr. 2023 Dec 1;12(1):e03237-23. doi: 10.1128/spectrum.03237-23 (PMC10783092; doi:10.1128/spectrum.03237-23)
Supplement: Tables S1 to S5 — All primers used in this research. [file spectrum.03237-23-s0001.pdf]

Table S1. RT-PCR primers used in this study

Primer name	Oligo (5'-3')	Use
16s RNA-F	AAGTTAATACCTTTGCTCATTGAC	Forward primer of *16s RNA* used to RT-PCR
16s RNA-R	GCTTTACGCCCAGTAATTCC	Reverse primer of *16s RNA* used to RT-PCR
marA RNA-F	ATCCGCAGCCGTAAGATGA	Forward primer of *marA* used to RT-PCR
marA RNA-R	GGTTCGGGTCAGAGTTTGTTG	Reverse primer of *marA* used to RT-PCR
acrA RNA-F	AAGATAGCGCGTAGGGTGATAGAC	Forward primer of *acrA RNA* used to RT-PCR
acrA RNA-R	GGCACGCTGAAACAAGAGAAC	Reverse primer of *acrA RNA* used to RT-PCR
acrB RNA-F	AAGAAGCCACCCGTAAGTCG	Forward primer of *acrB RNA* used to RT-PCR
acrB RNA-R	AGTAGAACCGCCAAAGAAGG	Reverse primer of *acrB RNA* used to RT-PCR

Table S2. *acrB* deletion primes used in this study

Name	Oligo (5'-3')	Use
acrB-P1	TCAGCCTGAACAGTCCAAGTCTTAACT TAAACAGGAGCCGTTAAGACATGATG GGAATTAGCCATGGTCC	Forward primer to obtain Km^R-FTR fragment with 50 bp *acrB* homology arms
acrB-P2	TTGCGCGGCCTTAGTGATTACACGTTG TATCAATGATGATCGACAGTATGGTGT AGGCTGGAGCTGCTTC	Reverse primer to obtain Km^R-FTR fragment with 50 bp *acrB* homology arms
acrB-H1	ATGGGAATTAGCCATGGTCC	Forward primer to check *acrB* gene deletion
acrB-H2	GTGTAGGCTGGAGCTGCTTC	Reverse primer to check *acrB* gene deletion

Table S3. pBBR-acrB construction primes used in this study

Name	Oligo (5'-3')	Use
pBBR-acrB-F	GTCGACGGTATCGATAAGCTTAA TGCCTAATTTCTTTATCGATCGC	Forward primer to generate PCR fragment used to construct complement *acrB* plasmid
pBBR-acrB-R	CTATAGGGCGAATTGGAGCTCTC AATGATGATCGACAGTATGGC	Reverse primer to generate PCR fragment used to construct complement *acrB* plasmid
pBBR-acrB-1	CGATAACCTGTGTCACCGTGT	Forward primer used to Verify whether a frame shift has occurred in pBBR-acrB
pBBR-acrB-2	CGCAAACCGCCTCTCCC	Reverse primer used to Verify whether a frame shift has occurred in pBBR-acrB
pBBR-acrB-YZ-F	AAATATTAACGCTTACAATTTCC ATTCGCCA	Forward primer used to verify the construction of pBBR-acrB
pBBR-acrB-YZ-R	TAAAGGTCTGATTGAAGCGACG CT	Reverse primer used to verify the construction of pBBR-acrB

Table S4. mutant *acrB* construction primes used in this study

Name	Oligo (5'-3')	Use
AcrB-A33W-37W-fwd	GCGGTGCAAT**CCA**AGGATATTG **CCA**CACCG	Forward primer to introduce amino acid change (GCG toACC, ACG toACC) at Ala33/Thr37 site in *acrB*
AcrB-A33W-37W-rev	TCGACGGTATCGATAAGCTTAA TGCCTAATTTCTTTATCGATCGC	Reverse primer to generate mutant *acrB* plasmid
AcrB-A100W-fwd	CGATATC**CCA**ATCAGTACCAGA CTCAA	Forward primer to introduce amino acid change (GCG to ACC) at Ala100 site in *acrB*
AcrB-A100W-rev	TGTGGCAATATCCTTGGATTGC ACCGCCGGCA	Reverse primer to generate mutant *acrB* plasmid
AcrB-N298W-fwd	ATTGCCGCAGCGGTATCCAGCG C**GGT**CGCACCGGTC	Forward primer to introduce amino acid change (AAC to TGG) at Asn298 site in *acrB*
AcrB-N298W-rev	TGGTACTGATGCGGATATCGCG CAGGTTCAGGTACA	Reverse primer to generate mutant *acrB* plasmid
acrB4-Fwd	TATAGGGCGAATTGGAGCTCTC AATGATGATCGACAGTATGGC	Forward primer to obtain *acrB* fragment to construct mutant *acrB*
acrB4-rev	CTGGATACCGCTGCGGCA	Reverse primer to obtain *acrB* fragment to construct mutant *acrB*
pBBR-fwd	AAGCTTATCGATACCGTCGACC	Forward primer to obtain linear pBBR1MCS-2 as a vector to construct mutant *acrB* plasmid
pBBR-rev	GAGCTCCAATTCGCCCTATAGT G	Reverse primer to obtain linear pBBR1MCS-2 as a vector to construct mutant *acrB* plasmid

Table S5. mutant *acrB* verification primes used in this study

Name	Oligo (5'-3')	Use
yz1	GAGCTCCAATTCGCCCTATAGTG	Forward primer used to verify mutations (A33W/T37W/A100W)
yz2	TAGTCGGAGATATCCTCCTGCGT	Reverse primer used to verify mutations (A33W/T37W/A100W)
yz3	ATGACCATGATTACGCCAAGCGC	Forward primer used to verify mutations (A100W/N298W)
yz4	AGATACATTACCAGGAACACGAGGATG	Reverse primer used to verify mutations (A100W/N298W)
